# The critical role of *S*-lactoylglutathione formation during methylglyoxal detoxification in *Escherichia coli*

**DOI:** 10.1111/j.1365-2958.2010.07426.x

**Published:** 2010-12

**Authors:** Ertan Ozyamak, Susan S Black, Claire A Walker, Morag J MacLean, Wendy Bartlett, Samantha Miller, Ian R Booth

**Affiliations:** School of Medical Sciences, University of AberdeenForesterhill, Aberdeen AB25 2ZD, UK

## Abstract

Survival of exposure to methylglyoxal (MG) in Gram-negative pathogens is largely dependent upon the operation of the glutathione-dependent glyoxalase system, consisting of two enzymes, GlxI (*gloA*) and GlxII (*gloB*). In addition, the activation of the KefGB potassium efflux system is maintained closed by glutathione (GSH) and is activated by *S*-lactoylGSH (SLG), the intermediate formed by GlxI and destroyed by GlxII. *Escherichia coli* mutants lacking GlxI are known to be extremely sensitive to MG. In this study we demonstrate that a Δ*gloB* mutant is as tolerant of MG as the parent, despite having the same degree of inhibition of MG detoxification as a Δ*gloA* strain. Increased expression of GlxII from a multicopy plasmid sensitizes *E. coli* to MG. Measurement of SLG pools, KefGB activity and cytoplasmic pH shows these parameters to be linked and to be very sensitive to changes in the activity of GlxI and GlxII. The SLG pool determines the activity of KefGB and the degree of acidification of the cytoplasm, which is a major determinant of the sensitivity to electrophiles. The data are discussed in terms of how cell fate is determined by the relative abundance of the enzymes and KefGB.

## Introduction

Bacteria have evolved elaborate and complex stress management strategies to minimize damage and thus, to enhance their survival during environmental changes (Booth, 2002). In addition, metabolic activity in itself can create significant stress, for example the production of hydrogen peroxide and oxygen radicals is a consequence of aerobic growth and the resulting oxidative damage requires both intrinsic and adaptive enzyme activities (Imlay, 2008; Korshunov and Imlay, 2010). Similarly, bacteria encounter electrophiles both as a metabolic consequence and as an environmental challenge. Among the most frequently encountered electrophiles is methylglyoxal (MG), which is produced by bacteria from sugars and amino acids and is believed to have a role in macrophage-mediated killing (Eskra *et al*., 2001; Eriksson *et al*., 2003; Ficht, 2003). MG is synthesized either from sugars by methylglyoxal synthase (MGS) (Totemeyer *et al*., 1998) or from threonine, serine and glycine by monoamine oxidase (Green and Lewis, 1968; Kim *et al*., 2004). In *Escherichia coli* the dominant route appears to be from sugars and arises when there is an accumulation of phosphorylated glycolytic intermediates above the level of 1,3-diphosphoglycerate and a lowering of the pool of inorganic phosphate (Hopper and Cooper, 1971; Ferguson *et al*., 1998; Totemeyer *et al*., 1998). MGS activity is determined by the balance between inorganic phosphate, which is a strong inhibitor, and dihydroxyacetone phosphate (DHAP), the substrate, which exhibits strong homotropic activation (Hopper and Cooper, 1971). Thus, production of MG only occurs when there is simultaneous depletion of phosphate and extremely high concentrations of DHAP, conditions that arise when sugar metabolism is strongly stimulated leading to excess carbon flow into the upper end of glycolysis (Freedberg *et al*., 1971; Ackerman *et al*., 1974; Burke and Tempest, 1990; Kadner *et al*., 1992; Russell, 1993). For *E. coli*, accumulation of MG above ∼0.3 mM in the medium results in growth inhibition and at levels above ∼0.6 mM the survival of cells is affected. Damage to DNA and to proteins has been observed (Krymkiewicz, 1973; Colanduoni and Villafranca, 1985; Ferguson *et al*., 2000) and both may contribute to cell death.

**Fig. 1:**
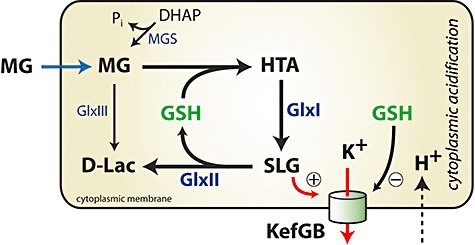
Detoxification of MG in enteric bacteria. Schematic representation of the major pathways of MG synthesis and detoxification, including the link to KefGB. d-Lac, d-Lactate; P_i_, inorganic phosphate.

Protection against electrophiles is multifactorial with contributions from glutathione (GSH), detoxification enzymes, DNA repair enzymes, peptide export systems and regulated K^+^ efflux systems (Ferguson and Booth, 1998; MacLean *et al*., 1998; Ko *et al*., 2005; Xu *et al*., 2006; Sukdeo and Honek, 2008). In *E. coli,* detoxification is primarily effected by the GSH-dependent glyoxalase system (GlxI and GlxII, products of the unlinked *gloA* and *gloB* genes) and their integration with the GSH adduct-gated KefGB K^+^ export systems (Fig. 1). Other enzymatic systems, particularly a range of oxidoreductases (Murata *et al*., 1989; Ko *et al*., 2005; Xu *et al*., 2006), may also play a role in detoxification. In the GlxI–II pathway, the substrate for GlxI is created by the spontaneous reaction between MG and GSH forming hemithioacetal (HTA). GlxI isomerizes this to *S*-lactoylGSH (SLG), which is the substrate for GlxII, a hydrolase. The final products are the relatively non-toxic molecule d-lactate and GSH, which is recycled in the cytoplasm. Although a GSH export system (Owens and Hartman, 1986; Pittman *et al*., 2005) has been identified, there is no evidence for a role in MG detoxification.

Protection by the KefGB and KefFC systems is dependent on their role in modulation of the cytoplasmic pH (Ferguson *et al*., 1995; 1996; 2000; Ferguson and Booth, 1998; MacLean *et al*., 1998). KefGB and KefFC are structurally related K^+^ efflux systems that are maintained inactive by the binding of GSH and are activated by binding of specific GSH adducts (Elmore *et al*., 1990; Ferguson *et al*., 1993; Miller *et al*., 1997; 2000; Roosild *et al*., 2009; 2010). Activation leads to rapid K^+^ efflux, which is quantitatively affected by several parameters: external K^+^ concentration, the activity of K^+^ uptake systems, the expression level of KefGB and KefFC and the intracellular concentration of the GSH adduct (Ferguson *et al*., 1993; 1996; MacLean *et al*., 1998; McLaggan *et al*., 2000). K^+^ efflux is accompanied by influx of H^+^ and Na^+^ (Bakker and Mangerich, 1982), but it is the lowering of the cytoplasmic pH that is critical for protection against MG (Ferguson *et al*., 1995; 2000). Lowering the cytoplasmic pH may slow the reaction of MG with guanine in DNA and with other macromolecules (Krymkiewicz, 1973).

We have previously proposed a model in which GlxI plays the major role in both determining the rate of MG detoxification and in modulation of KefGB activity by the intermediate SLG (Fig. 1) (MacLean *et al*., 1998). However, the original model could not be fully tested as mutants lacking GlxII could not be created and assays for SLG had not been developed. Thus the model rested on assumptions and essential elements were untested. Here, we report construction and characterization of GlxII mutants (Δ*gloB*). We integrate measurements of the SLG pools with assays of other parameters involved in MG detoxification and cell survival. In addition to supporting the original model, this comprehensive dataset demonstrate unequivocally the importance for survival of activation of KefGB and the consequent lowering of cytoplasmic pH. In particular we demonstrate that increased activity of the KefGB system can compensate for an impaired capacity to detoxify MG. The data are discussed in terms of the balance between GlxI, GlxII and the K^+^ efflux systems in determining the fate of individual cells.

## Results

### Modulation of GlxII activity

**Fig. 2:**
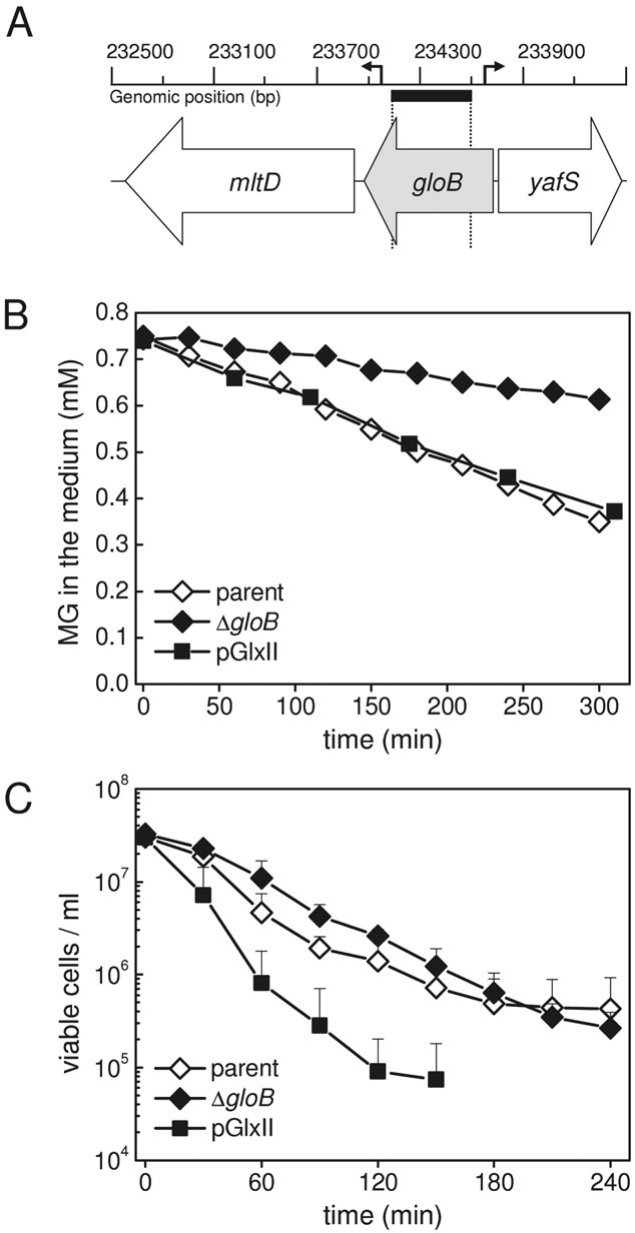
Mutant strain lacking GlxII exhibits a reduced capacity to detoxify MG, but is not more sensitive to the electrophile. A. Genomic context of the *gloB* gene in *E. coli*. The *gloB* gene (756 bp) encodes for the GlxII enzyme (EC 3.1.2.6, hydroxyacylglutathione hydrolase). The flanking genes *mltD* and *yafS* are transcribed divergently from *gloB*. Arrows indicate gene boundaries and transcriptional orientation. Genome co-ordinates are shown above the arrows. Bar and dashed lines indicate the deleted genomic region in the Δ*gloB* strain (MJF595). Arrows on the scale of the genome co-ordinates indicate promoter elements (−35 elements) for *mltD* and *yafS* as predicted by BPROM (see *Supporting information*). B. The *gloB* null mutant has impaired MG detoxification. Rate of MG detoxification does not change when GlxII is overexpressed. Cells from the parent (MJF274, ◊), Δ*gloB* (MJF595, ◆) and pGlxII (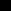) were grown to OD_650_ of ∼0.4 in K_0.2_ minimal media and diluted 10-fold into fresh media containing 0.7 mM MG. At intervals the medium was assayed for the disappearance of MG. The data are representative of three independent replicates. C. The *gloB* null mutant exhibits similar death kinetics to the parent strain upon MG stress. Cells overexpressing GlxII are more sensitive to MG. Cells from the parent (MJF274, ◊), Δ*gloB* (MJF595, ◆) and pGlxII (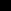) were grown exactly as in B and diluted into media containing 0.7 mM MG. Cell samples were taken at intervals and the number of viable cells determined. Data represent the mean of three independent replicates (standard deviations are shown).

To assess the importance of the GlxII activity and the activation of KefGB for survival upon exposure to MG we inactivated *gloB* (see *Experimental procedures*). Previous attempts to replace the *gloB* gene with antibiotic resistance cassettes (kanamycin and spectinomycin) were unsuccessful. We considered the possibility that replacement of the entire *gloB* gene might lead to polar effects with respect to the expression of the two genes on either side of *gloB*, namely *mltD* and *yafS*, which are separated by only 71 and 33 bp, respectively, from the *gloB* ORF (Fig. 2A). The *mltD* gene encodes for a membrane-bound lytic murein transglycosylase, which plays a major role in peptidoglycan expansion and recycling (Scheurwater *et al*., 2008; Suvorov *et al*., 2008). The *yafS* gene is believed to encode an *S*-adenosyl-l-methionine-dependent methyltransferase, but its physiological role remains unknown. From global array analysis under various growth conditions (GenExpDB, http://genexpdb.ou.edu), it is clear that both these genes are transcribed and thus either or both of these genes may be essential for cell function. Consequently we used a promoter prediction programme (Gama-Castro *et al*., 2008) (see *Supporting information*) for the design of the mutagenesis strategy. Based on this analysis, a 454 bp fragment (from 132 to 585) of the *gloB* structural gene was replaced (*Experimental procedures*) avoiding the putative promoter sequences for *mltD* and *yafS*. A short amino-terminal sequence of the GlxII protein (residues 1–43) may be expressed in the mutant strain MJF595 created in this study. However, from the crystal structure, this fragment is unlikely to form an enzymatically active protein as the critical metal and substrate binding sites are located in other regions (Zang *et al*., 2001; Campos-Bermudez *et al*., 2007).

The Δ*gloB* mutant grew at a similar rate to the parent in K_0.2_ minimal medium (Fig. S1A) and exhibited no obvious growth phenotype. Thus GlxII is not an essential enzyme during normal exponential growth. Some residual activity was detectable (corresponding to ∼6% of the parental GlxII activity), but was close to the analytical limit of the assay. The residual activity was not due to GlxI, as increasing the expression of this enzyme in the Δ*gloB* mutant did not increase the rate of breakdown of SLG (Table 1). Addition of 0.7 mM MG to both parent and mutant strains, in early exponential phase (OD_650_ = 0.05), caused immediate growth inhibition without recovery over the course of the experiment (Fig. S1A). MG disappeared from the medium in a linear fashion and, as expected, the rate was greatly reduced in Δ*gloB* cultures (Fig. 2B and 0.444 ± 0.015 µM MG min^−1^ and 1.155 ± 0.21 µM MG min^−1^, for mutant and parent respectively). We have previously observed a similar reduction in the capacity to detoxify MG in a Δ*gloA* null mutant (MacLean *et al*., 1998). However, cells retain the ability to breakdown MG, but at a much lower rate, which is consistent with the known presence of other enzymes that can metabolize MG (Misra *et al*., 1995; Misra *et al*., 1996).

**Table 1:** GlxII activity

Strain	Specific GlxII activity (U mg^−1^)
MJF274	0.069 ± 0.014
MJF595	0.004 ± 0.011
MJF274 pGlxII	1.789 ± 0.508
MJF274 pGlxI	0.067 ± 0.024

Enzyme activities were performed on three independent cytoplasmic cell extracts from each strain. For each extract the GlxII activity was measured using two different protein concentrations to ensure that the enzyme was rate-limiting. Activities increased proportionally with protein concentration and were averaged. The mean activity and standard deviation from independent extracts are shown. Strains: MJF274 (parent strain), MJF595 (Δ*gloB*), MJF274 pGlxII, MJF274 pGlxI (pMJM1).

The *gloB* gene, encoding for the GlxII enzyme, and its flanking regions were cloned into a moderate copy number vector to create pGlxII (see *Experimental procedures*). Transformation into *E. coli* MJF274 (parent strain) led to an approximately 25-fold amplification of GlxII activity in extracts from mid-exponential grown cells (Table 1). Cells expressing higher levels of GlxII grew at a similar rate to the parent strain in K_0.2_ minimal medium (Fig. S1B). Elevated synthesis of GlxII did not alter the rate of MG detoxification when cells were incubated with 0.7 mM MG (Fig. 2B). These data are consistent with previous observations that GlxI activity limits the rate of MG detoxification in parental cells (MacLean *et al*., 1998).

### Inactivation of *gloB* does not affect cell viability upon MG stress

Previously, we reported that a Δ*gloA* mutant, impaired in MG detoxification, exhibits increased sensitivity to MG, which can be explained by the persistence of the electrophile in the growth medium (MacLean *et al*., 1998). When mutant cultures (either Δ*gloA* or Δ*gloB*) were treated with 0.7 mM MG, the concentration remains above the lethal level (∼0.6 mM MG) for ∼6 h due to the slow rate of detoxification in the absence of the Glx pathway. In contrast, the parent strain detoxifies MG to non-lethal levels within ∼2–3 h (at low cell density, OD_650_∼0.05). Thus, it was expected that the Δ*gloB* mutant would exhibit a similar sensitivity to MG as the Δ*gloA* mutant. However, survival of the Δ*gloB* mutant during MG exposure was not impaired (Fig. 2C). Surprisingly, overexpression of GlxII increased sensitivity to MG despite having no effect on the rate of detoxification (Fig. 2B and C).

We further addressed if the viability phenotype of the Δ*gloB* mutant is a reflection of compensatory enzyme activities *in vivo*. FrmB and YeiG (EC 3.1.2.12) are major components of the formaldehyde detoxification pathway and have been reported to have low level hydrolytic activity against SLG (Gonzalez *et al*., 2006). The *yeiG* gene is transcribed constitutively whereas the *frmB* gene can be induced with formaldehyde (Gonzalez *et al*., 2006). Strains lacking GlxII and lacking either YeiG (MJF595 Δ*yeiG*) or FrmB (MJF595 Δ*frmB*) were created (*Experimental procedures*) and cell viability determined during exposure to 0.7 mM MG. The level of survival of both double mutants (Δ*gloB*–Δ*yeiG* or Δ*gloB*–Δ*frmB*) was indistinguishable from the Δ*gloB* mutant (Fig. S2), indicating that these systems do not have a physiologically significant role in MG detoxification.

### K^*+*^ efflux systems are hyperactive in a *gloB* null mutant

**Fig. 3:**
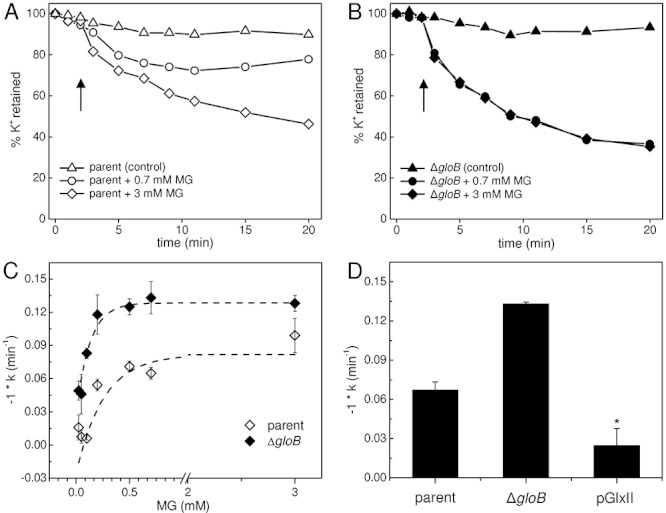
K^+^ efflux systems are hyperactive in a *gloB* null mutant. A and B. K^+^ efflux from the parent (MJF274, A) and Δ*gloB* (MJF595, B) upon exposure to different MG concentrations. Cells were grown to an OD_650_ of ∼0.8 in K^+^-rich minimal medium (K_115_), harvested and suspended in K^+^-free buffer. K^+^ efflux was measured in the absence (control; ▵, ▴) and in the presence of 0.7 mM (○, •) and 3 mM MG (◊, ◆). MG was added 2 min (indicated by arrow) after resuspension of cells in K^+^-free buffer. Control data were averaged for illustration. Data shown are representative of three independent replicates. At time zero MJF274 contained ∼494 ± 8 µmol K^+^ and MJF595 contained 485 ± 18 µmol K^+^ per gram dry cell weight. C. First order rate constants (*k*) for K^+^ efflux over a range of MG concentrations. K^+^ efflux from the parent (◊) and Δ*gloB* (◆) was measured using different MG concentrations (0.025–3 mM). Rate constants were determined over a period of 3 min after the addition of MG (*t*_2_ to *t*_5_; see also *Experimental procedures*) and multiplied by −1 for illustration purposes. Data represent the mean of three independent replicates (standard deviations are shown). Datasets for both strains were fitted using an exponential association function in the Origin 8.0 software {Equation: *y* = *y*_0_ + *A*_1_ × [1 − exp(−x/*t*_1_)] + *A*_2_ × [1 − exp(−x/*t*_2_)]} and the output for each dataset is shown as a dashed line. D. First order rate constants (*k*) for K^+^ efflux of the parent, Δ*glo*B and pGlxII when treated with 0.7 mM MG. Rate constants were determined as for Fig. 3C. *Not significantly different to the rate of spontaneous K^+^ loss from cells untreated with MG.

We have previously established that KefGB and KefFC are activated by electrophiles through the formation of GSH adducts (Elmore *et al*., 1990; Ferguson *et al*., 1993; 1995; 1997). From the study of a Δ*gloA* mutant we inferred that SLG was the metabolite activating KefGB during exposure to MG, as the HTA formed by a reversible reaction with GSH in such a mutant was insufficient to activate the K^+^ efflux system (MacLean *et al*., 1998). We predicted that a Δ*gloB* mutant, lacking GlxII activity, would accumulate SLG and thus, KefGB activity should be enhanced; conversely we predicted that overexpression of GlxII should diminish SLG pools and thus lower the rate of K^+^ efflux. Analysis of K^+^ efflux patterns in the parent strain MJF274 and the Δ*gloB* mutant MJF595, using a range of MG concentrations, supports this model. Accurate measurements of K^+^ efflux require cells to be incubated at higher cell densities than are used for growth and viability measurements (OD_650_∼0.8 for efflux assays compared with ∼0.05 for growth and viability). Consequently, in the parent strain, the MG concentration is continuously declining due to the high rate of detoxification in such conditions (0.7 mM MG falls to ∼0.3 mM in 30 min). Control experiments for cell viability and MG detoxification were carried out at high cell density under conditions identical to the measurements of cytoplasmic pH and K^+^ efflux (Fig. S3A and B). The rate constant for efflux was measured over the first 3 min, a period in which there was minimal lowering of the MG concentration (Fig. S3B). The rate and extent of K^+^ loss was faster in the Δ*gloB* strain than in the parent (Fig. 3). This effect was most marked at the lower MG concentrations and thus first order rate constants for the initial K^+^ efflux were measured at a range of concentrations (Fig. 3C). In the parent strain, the rate of efflux observed at very low MG concentrations (< 200 µM) was not significantly different from the rate of K^+^ loss in the absence of MG. For higher concentrations the rate constant increased and approached a maximum for concentrations ≥ 3 mM MG (Fig. 3C). In contrast, the Δ*gloB* mutant exhibited rapid K^+^ loss even at concentrations as low as 25 µM MG and the rate was not further stimulated by treatment with ≥ 200 µM MG (Fig. 3B and C). In cells overexpressing GlxII MG addition did not stimulate significant K^+^ efflux (Fig. 3D).

### Intracellular accumulation of SLG upon MG stress

**Fig. 4:**
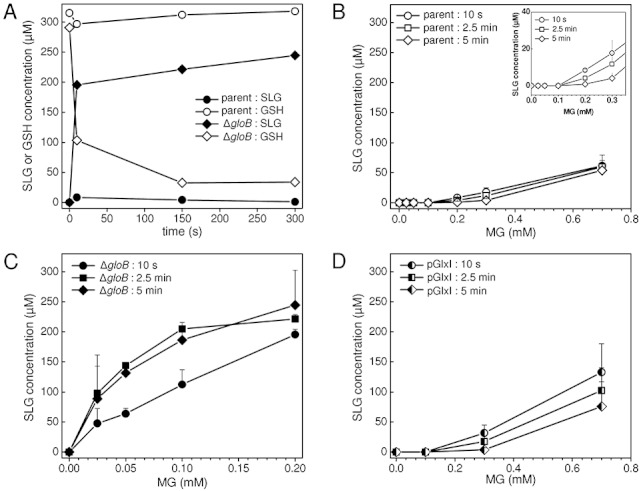
SLG accumulates rapidly to high levels in a *gloB* null mutant. A. Changes in GSH and SLG levels upon MG exposure were quantified in both the parent strain and the *gloB* null mutant. Cells were grown in K_0.2_ minimal medium to an OD_650_ of ∼0.8, handled as in K^+^ efflux assays and cells sampled at various time points. MG (0.2 mM) was added immediately after suspending cells in K_0_ buffer (*t*_0 s_). GSH (open symbols) and SLG levels (closed symbols) from the parent (○, •) and Δ*gloB* (MJF595, ◊, ◆) were quantified by LC-MS/MS. Data are representative of three independent replicates. Figures show metabolite concentrations as quantified in the extraction volume (see *Experimental procedures*); a concentration of 100 µM in the extraction volume equates to an intracellular concentration of ∼6.35 mM (see *Experimental procedures*). B–D. SLG levels in the parent (B), Δ*gloB* (C) and pGlxI (D) strains upon exposure to a range of MG concentrations. Cells were sampled 10 s (○, •), 2.5 min (□, 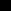) and 5 min (◊, ◆) after addition of MG, and the metabolite pools quantified by LC-MS/MS. The mean and standard deviation of three independent replicate experiments is shown.

We developed an LC-MS/MS assay to quantify intracellular GSH and SLG pools to obtain insight into the *in vivo* dynamics of SLG formation. Cells were grown in K_0.2_ minimal medium and GSH and SLG were extracted from cells with formic acid using a silicone oil centrifugation technique (see *Experimental procedures*). Pools of GSH and SLG were measured over the time period used to measure the rate constant for KefGB and using experimental conditions identical to those for the K^+^ efflux assays. GSH pools prior to MG addition were identical in the parent and Δ*gloB* strains within experimental error and no SLG could be detected prior to addition of MG (Table S1). Addition of MG (0.2 mM) caused a rapid increase in SLG, coincident with depletion of GSH, in both Δ*gloB* and parent; the increase was ∼20-fold greater in the mutant than the parent (Fig. 4A). In the parent, SLG pools were below the detection limit of the analytical technique at concentrations < 0.2 mM MG (Fig. 4B). At higher MG concentrations the SLG pool rose rapidly then declined slowly over the period of the assay (Fig. 4A). Pools of SLG in the Δ*gloB* strain also increased rapidly, even with only 25 µM MG, achieving ∼50% of the maximum value in 10 s, then climbed slowly over the next 2.5 min (Fig. 4C). Despite the absence of GlxII, complete conversion of the GSH pool to SLG was never observed, free GSH was always measurable ∼34 ± 4 µM (Table S1).

High SLG pools were also observed in strains overexpressing GlxI in which the highest SLG level was generally observed at 10 s and decreased thereafter (Table S1), consistent with the higher rates of detoxification observed in this strain (MacLean *et al*., 1998). SLG levels were significantly higher than in the parent strain (∼2.5-fold at 10 s, Fig. 4B and D). However, these did not reach the levels seen in the Δ*gloB* mutant, reflecting the continuing breakdown. Cells expressing high levels of GlxII did not accumulate significant SLG when incubated with MG (0.1–0.7 mM MG; Table S1).

### Relationship between SLG pool and KefGB activity

**Fig. 5:**
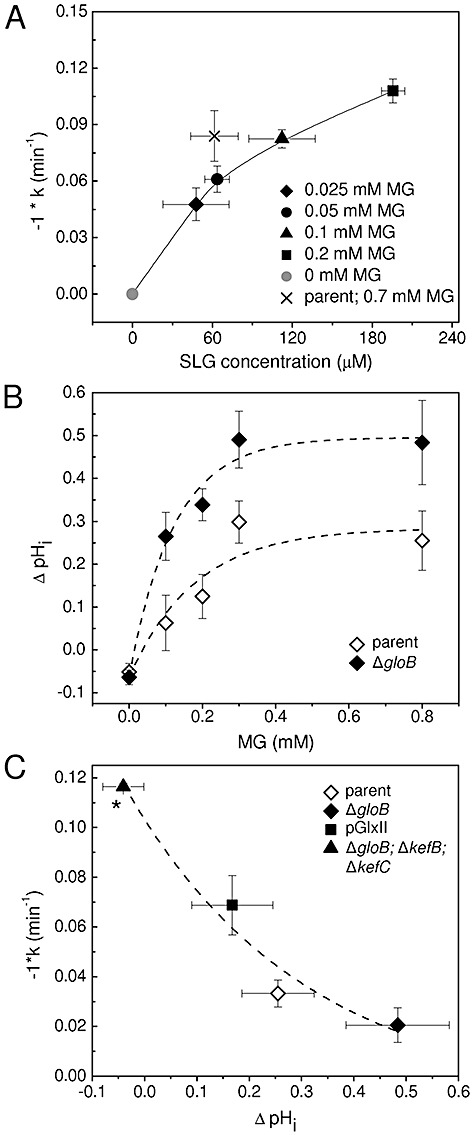
Complex relationships exist between SLG levels, activation of K^+^ efflux systems and modulation of pH_i_. A. First order rate constants for K^+^ efflux (*k*) from the Δ*gloB* mutant, and the parent were plotted against mean SLG levels at *t*_10 s_ after addition of selected MG concentrations. The data point in gray illustrates the lack of K^+^ efflux in the absence of SLG. B. The change in intracellular pH (ΔpH_i_) in the parent (◊) and Δ*gloB* (◆) as a function of the concentration of exogenously applied MG. The ΔpH_i_ was calculated as (*t*_10–14 min_) − (*t*_15–18 min_) where MG was added at *t*_15 min_ for each MG concentration. The data shown are means ± s.e.m. Datasets for both strains were fitted using an exponential association function in the Origin 8.0 software [Equation: *y* = *A*_1_ × exp(−x/*t*_1_) + *y*_0_] and the output for each dataset is shown as a dashed line. The adjusted *R*^2^ values for the parent and Δ*gloB* were 0.76 and 0.96 respectively. C. First order rate constants (*k*) for survival of the parent (◊), Δ*gloB* (◆), pGlxII (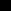) and Δ*gloB*; Δ*kefC*; Δ*kefB* (MJF596, ▴) upon MG exposure were derived from viable cell counts over the first 60 min after addition of 0.7 mM MG. These data were plotted against the ΔpH_i_ calculated as described in B. Data plotted are means ± s.e.m. Data points were fitted using an exponential association function in the Origin 8.0 software [Equation: *y* = *A*_1_ × exp(−x/*t*_1_) + *y*_0_]. The output is shown as a dashed line and the adjusted *R*^2^ = 0.89. *As a consequence of the method used to determine pH_i_ and subsequently derive ΔpH_i_, the absence of a drop in pH_i_ upon addition of MG, as is the case for MJF596, can lead to a negative value for ΔpH_i_ as the steady state pH_i_ measured over the time-course fluctuates around pH 7.8.

Measurements of the SLG pools and rate constant for K^+^ efflux under similar conditions allowed the determination of the adduct dependence of KefGB activity. The very slow MG breakdown in the Δ*gloB* strain allowed both the SLG pool and KefGB activity to be measured across a wide range of MG concentrations. Parallel measurements were made with the parent (Fig. 5A). The rate constant for efflux shows a non-linear dependence on SLG concentration (Fig. 5A). Data from the parent strain were consistent with this relationship (Fig. 5A). When excess MG was supplied (3 mM), which maximally activates KefGB, the rate constant was 0.1 ± 0.02 min^−1^ and 0.13 ± 0.01 min^−1^ for the parent and Δ*gloB* respectively (Fig. 3A and B). Remarkably, strong KefGB activation (to ∼25% maximum activity) was observed in the Δ*gloB* strain even with only 25 µM MG (Fig. 3C), which corresponded to a sixfold excess of GSH over SLG (Table S1). The precise relationship between KefGB activity and SLG pools is expected to be complex as the formation of SLG is accompanied by removal of the KefGB inhibitor, GSH.

### Greater cytoplasmic acidification is achieved in a *ΔgloB* mutant

The activity of the KefGB efflux system ultimately results in acidification of the cytoplasm, which limits the toxicity of MG (Ferguson *et al*., 1995; Ness and Booth, 1999). We investigated whether the increased activity of KefGB in the Δ*gloB* cells affected the cytoplasmic pH (pH_i_) upon MG stress. Experiments were conducted under the same conditions as for K^+^ efflux and SLG pool analysis. Cytoplasmic pH fell to a steady state level within 60 s of addition of MG and the change was dependent upon the presence of both KefGB and KefFC. Thus the pH change had different kinetics from K^+^ efflux, which continued for at least 15 min, albeit at progressively slower rates. The discrepancy between the change in pH and K^+^ loss is explained by previous studies that demonstrated that K^+^ efflux is accompanied by entry of both H^+^ and Na^+^ (Bakker and Mangerich, 1982). Cytoplasmic pH changed both as a function of the MG concentration and the strain (Fig. 5B) (a representative dataset showing the kinetics of the pH changes is presented in Fig. S4). There was no consistent difference in the cytoplasmic pH between the parent and the Δ*gloB* strain prior to addition of MG. After MG addition the cytoplasmic pH always fell to a lower value in the mutant, which is the predicted consequence of increased activity of KefGB (Fig. 5B). Overexpression of GlxII (strain MJF595 pGlxII) led to a small drop (∼0.1 pH units, data not shown). Overall, a simple correlation was found between the initial rate of loss of viability and the change in the steady state pH_i_ (Fig. 5C).

### The role of KefGB in protection against MG

**Fig. 6:**
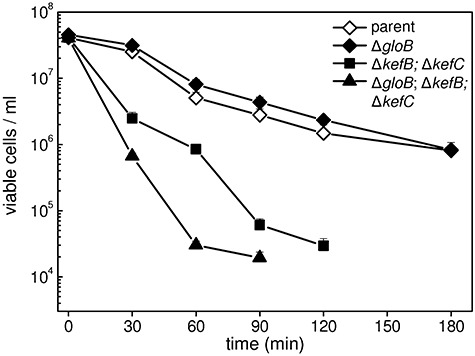
Survival of *gloB* null mutant upon MG stress depends on the activity of K^+^ efflux systems. Cells that lack the K^+^ efflux systems KefGB and KefFC, in addition to GlxII, are highly sensitive to MG exposure. Cells from the parent (◊), Δ*gloB* (◆), MJF276 (*kefB*, *kefC::Tn10*; 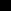) and MJF596 (Δ*gloB*, *kefB*, *kefC::Tn10*; ▴) were grown in K_0.2_ minimal media, exposed to 0.7 mM MG and viable cells enumerated exactly as for experiments presented in Fig. 2. The mean and standard deviation of three independent experiments is shown.

Our failure to observe increased sensitivity to MG in a Δ*gloB* strain (Fig. 2C) might be accounted for by the increased activation of KefGB and sustained lowering of the cytoplasmic pH (Figs 3C and 5C). We therefore constructed a strain lacking both KefGB (and KefFC) and GlxII (MJF596; *kefB*, *kefC::*Tn*10*, Δ*gloB*). The triple mutant grew at the same rate as the parent strain MJF274 in K_0.2_ minimal medium (data not shown) and was similarly impaired in MG detoxification as the *gloB* null mutant (0.463 ± 0.063 µM MG min^−1^; *n* = 3). Cells were very sensitive to MG (Fig. 6), despite their similar rate of detoxification to the strain lacking only GlxII. No viable cells were observed in the triple mutant after 2 h incubation with 0.7 mM MG. A mutant lacking both K^+^ efflux systems (MJF276; Δ*kefB*, Δ*kefC*) that retains detoxification was less sensitive to MG than the triple mutant, but cells were killed more rapidly than either the parent strain or the single Δ*gloB* mutant (Fig. 6). These data suggest that the efflux systems for K^+^ are more critical for survival than the detoxification pathway at low cell density.

## Discussion

The glyoxalase pathway provides an intrinsic advantage to cells when they are exposed to MG by catalysing the GSH-dependent conversion of MG to d-lactate, which can either be excreted or further metabolized. Mutants lacking GlxI exhibit a high sensitivity to MG; both growth inhibition at low MG concentrations (0.1 mM) and cell death at higher concentrations (0.7 mM) are increased approximately two fold (MacLean *et al*., 1998). In most detoxification pathways one would not necessarily look further than the loss of the capacity to remove the toxic molecule for an explanation of the phenotype. However, rapid cell death takes place in a time frame during which the MG concentration remains relatively constant. This is further supported by the Δ*gloB* mutant studied here, which does not share the increased sensitivity to MG seen in a Δ*gloA* strain despite similar continued exposure to high MG concentrations (Fig. 2). We have previously established that KefGB, but not KefFC, is strongly activated by metabolites derived from MG and GSH. Based on an analysis of the Δ*gloA* mutant and of strains possessing either KefGB or KefFC we proposed that KefGB was activated by the product of GlxI, SLG. The survival properties of the mutants studied here become explicable by this linkage and establish that the dominant factor determining survival is not detoxification but activation of the protecting K^+^ efflux system. Thus, Δ*gloA* (GlxI) and Δ*gloB* (GlxII) mutants exhibit almost identical rates of MG detoxification that are approximately threefold lower than observed in the parent (Fig. 2B, MacLean *et al*., 1998). However, in a GlxI mutant the only adduct formed is HTA, whereas in a GlxII mutant, SLG accumulates to high levels and the GSH pool is severely depleted (Fig. 4A; Table S1). GSH is an inhibitor of KefGB and HTA is only a weak activator, which would result in only limited activation of KefGB in a Δ*gloA* strain. In contrast, the enhanced pools of SLG and depletion of GSH in a GlxII mutant create optimal conditions for activation of KefGB (Fig. 3B and C) and result in a corresponding greater acidification of the cytoplasmic pH (Fig. 5B). Support for this model comes from our studies on the effect of the overexpression of GlxI or GlxII. The former stimulates detoxification, which suggests that this enzyme is limiting for the pathway. However, the stimulation is not proportional to the increase in enzyme activity, which is consistent with a fairly small gap between the relative activities of GlxI and GlxII, such that as GlxI activity is increased, GlxII becomes the limiting factor. Under these circumstances SLG accumulates (Table S1), with the effect of stimulating KefGB activity, which explains the dramatically increased protection against MG by overexpression of GlxI (MacLean *et al*., 1998). In contrast, overexpression of GlxII does not modify the detoxification rate, but sensitizes cells to MG (Fig. 2C). An increase in GlxII, in the context of a low but constant level of GlxI, depletes the SLG pool (Table S1) resulting in limited activation of KefGB and hence only moderate protection. Finally, the combination of mutations that eliminate KefGB, KefFC and GlxII renders cells as sensitive to MG as if they had a Δ*gloA* mutation (Fig. 6).

The relationship between the magnitude of the SLG pool, KefGB activity and pH_i_ is complex, but can be understood from the properties of the systems and the cell itself. The buffering capacity of the cytoplasm is at its lowest value around cytoplasmic pH values in the range pH 7–8 (Booth, 1985). Thus the cell is simultaneously at its most sensitive to perturbation of the environment due to limited buffering capacity, but also has the greatest potential to modulate cytoplasmic pH through changes in the balance of proton entry and efflux pathways. Previous work established that K^+^ efflux from *E. coli* is compensated by the entry of both H^+^ and Na^+^ in a ratio of approximately 1K^+^∼ 0.4H^+^ and 0.6Na^+^ (Bakker and Mangerich, 1982). The extremely rapid acidification of the cytoplasm upon activation of KefGB can be explained by initial rapid K^+^-linked H^+^ movements that are subsequently compensated by the reversibility of the Na^+^/H^+^ antiports (Padan *et al*., 2001) leading to exchange of cytoplasmic H^+^ for external Na^+^. Thus, the pH would be stabilized at a value set by the intrinsic properties of the KefGB system and the Na^+^/H^+^ antiports (NhaB and NhaA) (Dover and Padan, 2001; Padan *et al*., 2001). Our recent work (Roosild *et al*., 2010) shows that a single binding site on KefGB is shared by GSH and glutathione adducts. Activation of KefGB by MG requires displacement of GSH and binding of SLG. The cytoplasmic pool of GSH is suggested to be ∼20 mM (McLaggan *et al*., 2000; Fahey, 2001; Bennett *et al*., 2009). We observed that activation of KefGB in a Δ*gloB* strain requires only low concentrations of SLG, even in the presence of a sixfold excess of GSH (Figs 4A and 5A). Even in the parental strain, SLG is always at a lower concentration than GSH and consequently, the activation of KefGB is not maximal at the normal levels of abundance of GlxI and GlxII. Thus, the change in cytoplasmic pH is constrained by the relative abundance of GlxI and GlxII activities and the concentration of MG.

The data presented here point to an important relationship between the intrinsic activity of GlxI, GlxII and KefGB. The effects of GlxI overexpression indicate that although the enzyme is, on a population basis, limiting for the rate of detoxification. A 30-fold increase in GlxI activity produces only a twofold enhancement of the detoxification rate (MacLean *et al*., 1998) (see also Fig. S3B). From this we infer that GlxI and GlxII activities in cells are similar, such that large increases in GlxI cannot be manifested as increased detoxification rates due to the limitation that is imposed by GlxII activity. Given that most detoxification enzymes are expressed at low levels in the cell there is considerable scope for cell-to-cell variation in enzyme level. GlxI and GlxII are encoded by separate unlinked genes and there is no co-ordination of their expression. Consequently, in each cell their abundance should vary independently. We have calculated the abundance (in cells grown to mid-exponential phase in minimal medium) to be ∼130 GlxI and ∼1500 GlxII molecules per cell (see *Supporting information*). Stochastic variation of protein abundance will have a greater impact on GlxI and thus some cells will have a balance that favours SLG accumulation (high GlxI: GlxI > GlxII) whereas others (low GlxI: GlxII > GlxI) will have lower cytoplasmic pools of this adduct. Thus, on an individual cell basis, some cells will experience greater protection than others. The independence of the expression of KefGB from the detoxification enzymes creates a further dimension of variability that is superimposed on the modulation of SLG pools. This means that some cells may gain further protection; the optimal solution would be simultaneous enhanced levels of GlxI and KefGB, coupled with low activity of GlxII. In this way the cell will experience maximum protection coupled with detoxification.

In this study we have defined the relationship between sensitivity to MG and the relative activities of the major detoxification pathway, GlxI–II, and the KefGB K^+^ efflux system. The study demonstrates the benefits to the cell of linking an intermediate in detoxification to the activation of the protective K^+^ efflux system. Small changes in detoxification enzymes determine the maximum activity of KefGB. It is perhaps counterintuitive that cells possess protective capacity that is not always fully utilized. At low cell density detoxification capacity is secondary to ion channel activity in determining single cell fate. Conversely at high cell densities detoxification capacity is the primary determinant of cell survival. Stochastic distribution of the proteins between cells in a population ensures that a few cells are highly protected and survive exposure to MG. In addition, it seems plausible that avoiding maximum stimulation of KefGB in all cells prevents excessive acidification that might be itself detrimental. The paradox is that in wild-type populations the majority (99.9%) of cells are terminally damaged (i.e. unable to form colonies after dilution and plating on fresh medium) but continue to contribute to MG removal. Thus, the survivors are aided by the dead and dying cells.

## Experimental procedures

### Strains and plasmids

All experiments were performed with *E. coli* K-12 derivative strains and are listed in Table 2. See further below for details of strains and plasmids created in this study.

**Table 2:** List of strains and plasmids

Strain	Genotype	Source/reference
MJF274	*F^-^*, *thi, rha, lacZ, kdpABC5, lacI, trkD1*	Ferguson *et al*. (1993)
MJF276	MJF274, *kefB*, *kefC::*Tn*10*	Ferguson *et al*. (1993)
MJF595	MJF274 Δ*gloB<>cm*	This study
MJF596	MJF276 Δ*gloB<>cm*	This study
MJF625	MJF595 Δ*yeiG::kan*	This study
MJF626	MJF595 Δ*frmB::kan*	This study
DY330	W3110 Δ*lacU169gal490 pgl*Δ*8 λcI857*Δ*(cro-bioA)*	Yu *et al*. (2000)
JW2141	BW25113 Δ*yeiG::kan*	Baba *et al*. (2006)
JW0346	BW25113 Δ*frmB::kan*	Baba *et al*. (2006)
Plasmid	Description	Source/reference
pHG165	pBR322 copy number derivative of pUC8	Stewart *et al*. (1986)
pMJM1	A pHG165 derivative into which *gloA* was cloned and is transcribed from its own promoter.	MacLean *et al*. (1998)
pGlxII	A pHG165 derivative into which *gloB* was cloned and is transcribed from its own promoter.	This study

### Growth media

For physiological assays cells were grown either in K_0.2_ minimal medium containing ∼0.2 mM K^+^ or K_115_ minimal medium containing ∼115 mM K^+^ (Epstein and Kim, 1971) depending on the experimental design. Both media were supplemented with 0.2% (w/v) glucose, 0.0001% (w/v) thiamine, 0.4 mM MgSO_4_ and 6 µM (NH_4_)_2_SO_4_·FeSO_4_. LK complex medium (Rowland *et al*., 1985) was used for cell growth for DNA manipulations. In the case of strains carrying an antibiotic resistance marker, no antibiotics were used in physiological assays as the routinely used resistance determinants were stable and were not lost in the absence of antibiotics. Solid media contained 14 g l^−1^ agar. To prepare solid K_0.2_ medium the agar was washed with a 1 M NaCl solution to displace trace amounts of K^+^ and then washed several times with distilled water before use.

### Cloning of the *gloB* gene

The *gloB* gene was PCR-amplified using whole cells of MJF274 as a template. Primers GloBI (5′-CAACCAGCGTCGACTGTAC) and GloBII (5′-GGTATCACCCAGTGGATCC) were designed 1.1 kb downstream and 1 kb upstream of *gloB* respectively. The primers contained HincII and BamHI restriction sites respectively (underlined). The amplified 2.8 kb product was digested with HincII and BamHI restriction enzymes. The cloning vector pHG165 was digested with HindIII, followed by treatment with Klenow enzyme to fill the HindIII site. Subsequently, the vector was digested with BamHI. The PCR product was ligated into the vector to create plasmid pGlxII from which the *gloB* gene is expressed from its native promoter.

### Construction of *gloB* null mutants

The *gloB* gene was disrupted with a chloramphenicol resistance cassette (Cm^R^) by homologous recombination mediated by λ-phage functions (recombineering). Recombineering primers *gloB KO-A* (5′-TGCCGCTCATTTTTCAGAATTACGGGTAGTGTTATTTGATTTTTTGCCCGACCAGCAATAGACATAAGCG) and *gloB KO-B* (5′-GATCCCGGAGACGCAGAGCCAGTATTAAACGCCATTGCCGCCAATAACTGATAAATAAATCCTGGTGTCCC) contained homologous sequences to the 3′ and 5′ of the *gloB* gene respectively. The incorporation of the Cm^R^ was predicted to create a fused ORF with the remaining 5′*gloB* sequence. Therefore, a double stop codon was incorporated into primer *gloB KO-B* (underlined) to prevent possible expression of a fusion protein driven by a *gloB* promoter. A 454 bp fragment (from 132 to 585) of the structural gene, where 1 is the first bp of the start codon, was replaced by the Cm^R^ cassette. Hence a short sequence of the GlxII protein may be expressed. The Cm^R^ cassette was amplified by colony-PCR from a strain carrying the resistance marker (MG1655 *zwf<cm>*; kindly provided by Michelle Brewster, SAIC, Frederick, USA) using the recombineering primers above. Recombineering was performed using strain DY330 and essentially as described by Yu *et al*. (Yu *et al*., 2000). Putative *gloB* integrants showed normal colony morphology and were purified twice on LK solid media containing 10 µg ml^−1^ chloramphenicol to check for the stability of the resistance marker. Recombination at the correct locus was investigated by PCR analysis using a primer pair flanking the *gloB* gene (*gloB KO check F*: 5′- CGCTTCGCAATTGATTTC; *gloB KO check R*: 5′-GGCGAGTAATATCGCTTT). The mutation was further confirmed by DNA sequencing of the PCR product across the borders of the site of recombination using primers *gloB KO check F and R* and *Cm Seq 1* (5′-CTTCATTATGGTGAAAGTTGG) and *Cm Seq 3* (5′-GATAATAAGCGGATGAATGG) located within the Cm^R^ cassette thus verifying successful creation of strain DY330 *gloB<>cm*[*<>* indicates replacement by recombineering approach (Yu *et al*., 2000)]. The mutation was transduced into strain MJF274 by P1 transduction creating strain MJF595. An overnight culture of strain DY330 *gloB<>cm* was grown in LK medium at 32°C (shaking at 250 r.p.m.). The culture was diluted 100-fold into fresh LK medium also containing 0.2% glucose, 10 mM MgCl_2_ and 5 mM CaCl_2_. Cells were grown at 32°C with shaking until minor growth was apparent when P1 phage lysate was added. The cultures were incubated further until cell lysis was evident. Lysates were treated with chloroform (50 µl per 10 ml lysate) and incubated at 37°C for 30 min without shaking. Cell debris was removed by centrifugation at 4300 *g* for 15 min and the supernatant passed through a filter (Whatman PURADISC™ filter, 0.25 µm) into sterile tubes containing chloroform (20 µl per 5 ml lysate). Lysates were either used immediately to infect a recipient strain (see below) or kept at 4°C for long-term storage. To create strain MJF595 (*gloB<>cm*) an overnight culture of MJF274 was grown in LK medium at 37°C (shaking at 250 r.p.m.). Aliquots (200 µl) were transferred into microcentrifuge tubes and cells harvested at 12000 r.p.m. for 30 s. Cells were suspended in 100 µl P1 salts solution (100 mM MgCl_2_ and 5 mM CaCl_2_) and a range of volumes of P1 donor lysate (see above) were added. Tubes were incubated at 37°C for 30 min. Subsequently cells were centrifuged at 12 000 r.p.m. for 30 s and suspended in 0.5 ml LK containing 20 mM sodium citrate (LK-NaCitrate). Cells were pelleted again as above and suspended in 1 ml LK-NaCitrate and incubated at 37°C for 1 h. Cells were pelleted and suspended in 100 µl LK-NaCitrate and plated onto selective LK-NaCitrate plates containing chloramphenicol (10 µg ml^−1^). Cells were incubated overnight at 37°C and transductants were purified twice in succession on selective LK-NaCitrate plates. The *gloB<>cm* mutation was also transduced into strain MJF276 by P1 transduction to create strain MJF596 (Δ*kefB*, Δ*kefC*, Δ*gloB<>cm*). Strains MJF625 and MJF626 were made by transducing MJF595 with P1 donor lysates prepared from strains JW2141 and JW0346, respectively, thus creating strains that lack known enzymes with minor *S*-lactoylglutathione hydrolase activity in addition to GlxII.

Interestingly, we observed some differences in the transfer of the *gloB* null mutation into different *E. coli* strains by P1 transduction. The *gloB<>cm* mutation, initially created in strain DY330, could be transduced into strain MJF274, as described above, and transductants were obtained after overnight incubation (∼16 h). In contrast, upon transfer of the mutation into strain MG1655, transductants were only evident after prolonged incubation (∼40 h) despite similar growth rates of both parent strains. Notably, upon purification, the MG1655 derivative strain grew at a similar rate as the parent strain and had no obvious phenotype (data not shown) possibly indicating the acquisition of a secondary mutation allowing cells to recover normal growth.

### K^+^ efflux assays

K^+^ efflux from cells was measured as described previously (Elmore *et al*., 1990; Ferguson *et al*., 1993). Briefly, cells were grown to late logarithmic phase (OD_650_∼0.8) in K_115_ medium, collected by filtration onto a cellulose acetate membrane (0.45 µm), washed with K_0_ buffer containing ∼5 mM (K_5_ buffer) and suspended in K_0_ buffer. Cells were rapidly transferred into thermally insulated glass pots and kept at 37°C under continuous stirring. Samples (1 ml) were taken at various time points, cells pelleted in a microcentrifuge at 14 000 r.p.m. for 30 s, the supernatant quickly aspirated and the K^+^ content of the cells determined by flame photometry after lysis by boiling in distilled water. MG (Sigma, M0252) was added from stock solutions to the test suspension 2 min after suspending cells in K_0_ buffer. The first order rate constants for K^+^ efflux (*k*) were calculated by transforming the values of K^+^ levels to the natural logarithm and by determining the slope of the decline in the linear range after addition of MG. For illustration purposes the K^+^ levels were normalized to the value at *t*_0 min_, defined as 100%.

### Cell viability and MG detoxification assays

Overnight cultures were grown in K_0.2_ medium and diluted into fresh, pre-warmed K_0.2_ medium to an OD_650_ of ∼0.05. Cells were grown to mid-exponential phase (OD_650_∼0.4) and diluted 10-fold into pre-warmed K_0.2_ medium also containing MG. Samples were taken at various time points and cell viability and MG detoxification assays performed as described previously (Ferguson *et al*., 1993; Totemeyer *et al*., 1996). Viable cells were recovered on solid K_0.2_ media plates.

### Preparation of cytoplasmic cell extracts and GlxII enzyme assays

Cells were grown in K_0.2_ medium to mid-exponential phase (OD_650_∼0.4) exactly as for the assays above; however, they were harvested at this point by centrifugation at 4300 *g*. Cells were washed, suspended in 50 mM potassium phosphate buffer (pH 6.6) and disrupted by two passages through a French press at 18 000 Psi. Bulk cell debris and membrane fractions were removed by centrifugation at 4300 *g* and subsequent centrifugation at 110 000 *g*. Cytoplasmic cell extracts were stored at −20°C until enzyme assays were performed. Protein quantification was performed using the Lowry assay (Lowry *et al*., 1951). GlxII activities were measured as a modification of a previously described method (Racker, 1951; Oray and Norton, 1982), in which SLG hydrolysis is measured by the decrease in A_240_. Enzyme assays were performed in 50 mM potassium phosphate buffer (pH 6.6) at 37°C using a Shimadzu UV 2101PC spectrophotometer. The reaction mixture contained 1 mM SLG (Sigma, L7140) in a total volume of 0.4 ml, using a 0.1 cm path length spectrophotometer cuvette. Enzyme activity was expressed as units per cytoplasmic cell protein (U mg^−1^) using a molar extinction coefficient of 3060 M^−1^ cm^−1^ (Racker, 1951), where 1 unit is defined as the amount of enzyme catalysing the formation of 1 µmol min^−1^ SLG. Enzyme activities were determined using two different enzyme concentrations to ensure that the enzyme was rate-limiting.

### Determination of intracellular GSH and SLG levels by LC-MS/MS

The choice of the experimental design to extract the metabolites from the cells was guided by the need to correlate SLG levels directly with K^+^ efflux. Therefore, cells were grown and treated as in K^+^ efflux experiments as described above except that cells were grown in K_0.2_ medium. After suspension of cells in K_0_ buffer 1 ml samples were taken at various time points before and after exposure to MG and transferred into previously prepared microcentrifuge tubes. Microcentrifuge tubes contained 40 µl 2.5 M formic acid (Sigma, 251364) with 50 µM Glu-Glu (Sigma, G3640, as an internal standard for LC-MS/MS analysis), overlayed with 500 µl silicone oil mixture prepared from silicone oils of different densities (AR20: Fluka, 10836; AP100: Fluka, 10838; proportion of 3:2). The sample tubes were centrifuged at 14 000 r.p.m. for 30 s. Cells that passed through the silicone into the formic acid were permeabilized and thus all cell reactions ceased immediately. Medium and silicone oil were removed by vacuum aspiration and the formic acid, with the cell debris, was transferred into fresh microcentrifuge tubes. The samples were centrifuged at 14 000 r.p.m. for 15 min at 4°C, the supernatants removed to separate tubes and stored at −20°C. Subsequently, samples were analysed by LC-MS/MS using quantification based on standard curves for GSH (Sigma, G6529) and SLG (Sigma, L7140) prepared on the same day as the cellular samples by adding appropriate volumes from frozen stocks to the formic acid/Glu-Glu extraction solution. The LC-MS/MS was performed using a Thermo Surveyor-TSQ Quantum system with electrospray ionization in the positive ion mode. A Stability BSC 17 (5 µ) column (150 × 2 mm) was used and the analytes eluted with a mobile phase comprising: 50% 7.5 mM ammonium formate, pH 2.6 (formic acid) and 50% acetonitrile at a flow rate of 0.2 ml min^−1^. The column was maintained at 45°C. Electrospray ionization conditions were as follows: spray voltage 4 kV, sheath gas pressure 60, auxiliary gas 0 and capillary temperature 375°C. Detection was carried out in SRM mode at a collision pressure of 1.4 and a collision energy of 13 V using the following SRM transitions: GSH m/z 308 – m/z 179, SLG m/z 380 – m/z 233 and Glu-Glu (internal standard) m/z 277 – 241. Quantification was performed using Xcalibur software. All samples and standards were diluted 1:100 with water prior to injection (1 µl) and were maintained at 4°C in the autosampler. It was not possible to resolve SLG and GSH chromatographically; however, parent masses and fragment masses were suitably different to allow discrimination by the subsequent MS. Control experiments with a *gloA* null mutant verified that the LC-MS/MS assay did not detect the isomer of SLG, namely HTA. HTA is the product of the chemical equilibrium between MG and GSH (Fig. 1). The equilibrium of this reaction is in favour of HTA, which is then converted to SLG by the action of the GlxI enzyme. Note that, in this assay, measured GSH levels do not necessarily reflect *in vivo* GSH levels during the MG detoxification process because of the chemical equilibrium with HTA. Measured GSH levels will be a composite of actual GSH levels and GSH that was conjugated as HTA at the time of cytoplasmic extraction. The HTA molecule is unstable upon disturbance of the equilibrium with GSH + MG, i.e. upon dilution of the cytoplasmic cell volume in formic acid the equilibrium is driven back to GSH and MG. Furthermore, metabolite concentrations presented in this study are the levels as quantified in the extraction volume (40 µl formic acid). An approximation of intracellular concentrations can be derived from the relationship between OD_650_ and cytoplasmic volume [1 ml cell culture at OD_650_ 2 = ∼1.6 µl cytoplasmic volume; (Kroll and Booth, 1981)]. Thus, the total cytoplasmic volume in the assay is ∼0.64 µl (8 × 10^8^ cells), and a concentration of 100 µM in the extraction volume equates to an intracellular concentration of ∼6.35 mM.

### Measurement of intracellular pH

The magnitude of the pH gradient was estimated from the distribution of a weak acid, and pH_i_ (internal pH) calculated from knowledge of pH_o_ (external pH). Cells were grown in K_0.2_ minimal medium until they reached an OD_650_∼0.8. The cell suspension was transferred into two thermally insulated glass pots (37°C) and ^14^C benzoic acid (4.5 µM final concentration; specific activity 0.1 µCi ml^−1^) and ^3^H-water (specific activity 1 µCi ml^−1^), as marker of extracellular water, were added to the cultures (Kroll and Booth, 1981). After 5 min incubation, 1 ml samples were taken at timed intervals and the cells and supernatant separated by centrifugation (14 000 r.p.m. for 20 s). For each sample, 100 µl of the supernatant was transferred to a scintillation vial containing 200 µl of cell suspension that had not been treated with radioactivity; the remaining supernatant was aspirated from the cell pellet and discarded. Cell pellets were suspended in 200 µl K_0.2_ buffer containing 0.2% glucose and the suspension transferred into a scintillation vial containing 100 µl of the same buffer. Samples of supernatant and pellet were counted for radioactivity on a preset ^3^H/^14^C program of a Tri-Carb 2100 TR liquid scintillation analyser. The pH gradient and subsequently the pH_i_ were calculated as described previously (Booth *et al*., 1979).
