# Supplementary material for: The critical role of S-lactoylglutathione formation during methylglyoxal detoxification in Escherichia coli
Source: Mol Microbiol. 2010 Dec;78(6):1577–90. doi: 10.1111/j.1365-2958.2010.07426.x (PMC3412212; doi:10.1111/j.1365-2958.2010.07426.x)
Supplement: Supplementary file 1 [file mmi0078-1577-SD1.pdf]

Supplementary information

SLG and GSH pools

The SLG and GSH pools were measured for the parent strain, Δ*glo*B, pGlxI and pGlxII at 0, 10, 150 and 300 s intervals following treatment with a range of MG concentrations (Table S1 A and B). The mean and standard deviations are shown. The values given for SLG and GSH concentrations are the final concentrations after extraction from ~ 0.8 $x10^9$ cells in 40 µl formic acid. An estimation of intracellular SLG and GSH concentrations can be derived from the knowledge of the relationship between OD and cytoplasmic volume determined as 1ml cells at OD_{650} = 2 has a cytoplasmic volume of 1.6 µl (Ahmed and Booth 1981).

A

		SLG (µM) at timed intervals (s)			
	MG [mM]	0	10	150	300
Parent	0	ND*	ND	ND	ND
	0.025	ND	ND	ND	ND
	0.05	ND	ND	ND	ND
	0.1	ND	ND	ND	ND
	0.2	ND	8.38 ± 0.48	4.14 ± 3.59	0.86 ± 1.49
	0.3	ND	17.73 ± 7.03	11.71 ± 5.88	4.05 ± 2.8
	0.7	ND	61.48 ± 17.84	59.87 ± 10.86	53.96 ± 10.26
Δ *glo*B	0	ND	ND	ND	ND
	0.025	ND	47.77 ± 24.84	97.73 ± 63.83	88.76 ± 54.38
	0.05	ND	63.56 ± 9.27	143.95 ± 4.14	131.58 ± 9.16
	0.1	ND	112.32 ± 24.88	204.91 ± 10.93	186.46 ± 17.92
	0.2	ND	195.62 ± 8.73	221.44 ± 7.37	244.68 ± 58.24
pGlxI	0	NA†	NA	NA	NA
	0.1	ND	ND	ND	ND
	0.3	ND	31.65 ± 13.51	17.73 ± 8.92	3.95 ± 3.37
	0.7	ND	132.85 ± 47.42	102.38 ± 37.82	75.81 ± 41.27
pGlxII	0	NA	NA	NA	NA
	0.1	ND	ND	ND	ND
	0.3	ND	ND	ND	ND
	0.7	ND	1.73 ± 3	1.5 ± 2.59	1.57 ± 2.72

B		GSH (μM) at timed intervals (s)			
	MG [mM]	0	10	150	300
Parent	0	280.77 ± 62.23	318.40 ± 58.68	307.07 ± 67.2	317.95 ± 43.03
	0.025	298.18 ± 27.88	289.7 ± 31.28	331.09 ± 19.02	345.47 ± 28.51
	0.05	317.15 ± 32.55	336.4 ± 34.24	353.61 ± 39.66	369.25 ± 46.09
	0.1	302.71 ±70.24	305.04 ± 65.15	337.21 ± 62.74	332.9 ± 64.75
	0.2	315.26 ± 64.17	296.76 ± 50.15	312.01 ± 62.57	318.05 ± 55.01
	0.3	273.35 ± 50.94	228.25 ± 13.89	223.28 ± 8.11	255.91 ± 33.31
	0.7	246.07 ± 32.6	169.38 ± 33.07	168.83 ± 33.36	182.51 ± 38.27
Δ *gloB*	0	321.14 ± 37.36	315.01 ± 19.57	313.96 ± 11.23	303.88 ± 14.02
	0.025	188.64 ± 167.34	220.98 ± 36.96	169.81 ± 51.04	163.4 ± 61.94
	0.05	311.05 ± 68.66	250.13 ± 37.22	153.22 ± 45.18	141.35 ± 36.12
	0.1	294.36 ± 13.72	157.81 ± 6.95	64.61 ± 15.32	55.92 ± 11.08
	0.2	290.93 ± 7.75	103.52 ± 2.59	32.7 ± 5.72	33.89 ± 3.68
pGlxI	0	NA	NA	NA	NA
	0.1	252.64 ± 50.08	240.85 ± 61.25	272 ± 52.63	255.74 ± 51.24
	0.3	231.74 ± 28.92	214.56 ± 38.15	204.73 ± 12.33	223.05 ± 39.06
	0.7	269.11 ± 33.47	120.68 ± 30.12	132.7 ± 10.12	153.84 ± 23.03
pGlxII	0	NA	NA	NA	NA
	0.1	275.06 ± 16.02	274.4 ± 20.25	303.84 ± 12.83	293.44 ± 8.92
	0.3	231.66 ± 21.26	250.08 ± 20.31	264.96 ± 57.97	262.32 ± 43.47
	0.7	260.30 ± 23.73	261.42 ± 98.67	258.01 ± 15.77	272.74 ± 14.46

* Not detected † Not assayed

Promoter predictions for mltD and yafS

To guide our experimental approach in creating a *gloB* null mutant, an assessment of promoter elements for the respective genes was undertaken. In the first instance, promoter predictions were accessed on RegulonDB, a curated database containing information on the transcriptional regulatory network of *E. coli* K-12 (Gama-Castro *et al.*, 2008). At the time of preparation of this paper RegulonDB stated four potential σ^{70} promoters for *mltD* with the furthest predicted -35 element being 184 bp upstream of the start codon (Table S2) and thereby within the *gloB* gene. No promoter predictions were stated for *yafS* on the RegulonDB database. This analysis was complemented using the web-based tool BPROM that can predict σ^{70} promoters (www.softberry.com). The upstream sequences (500 bp from

protein encoding sequence) of *mltD* and *yafS* were analysed with BPROM using the default settings and potential -10 and -35 promoter elements identified. BPROM predicted one promoter region for *mltD*, located up to 180 bp upstream (between positions 234109 and 234135 on the chromosome, Table S3) thereby overlapping with the furthest promoter prediction stated in RegulonDB. Two putative promoter regions were predicted for *yafS* by BPROM (Table S4). The -35 element of the first region was located 68 bp (end of -35 box) upstream from the start codon and thereby also within the *gloB* gene. A second promoter was predicted further away (~450 bp from start codon), however, this was not considered in our strategy to inactivate *gloB* since this may have resulted in the expression of a considerable GlxII fragment.

Table S2. Computational σ^{70} promoter predictions for *mltD* as stated on RegulonDB.

Promoter name	Position +1	Box -35	Spacer between -10 & -35 box	Box -10	Score
*mltD*p1	234025	GTTTTGCAT	17	GATAGGTT	5.07
*mltD*p2	234041	AACCTGAAG	13	GTTAAGGT	7.33
*mltD*p3	234074	TAATTAATG	16	ATTATTGC	2.54
*mltD*p4	234100	TTTTTTTAA	16	TTTAATTA	3.72

See the following web address for more details:
http://regulondb.ccg.unam.mx/

Table S3. σ^{70} promoters for *mltD* as predicted by BPROM

1) Length of sequence:	500		
2) Threshold for promoters:	0.20		
3) Number of predicted promoters:	1		
4) Promoter Position:	354		LDF: 7.12
-10 box at position	339	TGATTTAAT	Score 44
-35 box at position	321	TTTAAG	Score 35

Table S4. σ^{70} promoters for *yafS* as predicted by BPROM

1) Length of sequence:	500		
2) Threshold for promoters:	0.20		
3) Number of predicted promoters:	2		
4) Promoter Position:	144		LDF: 5.21
-10 box at position	129	TCCTAAAGT	Score 43
-35 box at position	108	TTTACT	Score 42
Promoter Position:	470		LDF: 3.82
-10 box at position	455	TGTTAAGAT	Score 70
-35 box at position	433	TTGTCA	Score 53

Explanations for BPROM outputs:

1) The length of presented sequence.
2) LDF threshold (default).
3) The number of predicted promoters.
4) The positions of predicted promoters and their scores with 'weights' of two conserved promoter boxes. Promoter position is assigned to the first nucleotide of the transcript (transcription start site position).

See the following web address for more details:
http://linux1.softberry.com/berry.phtml?topic=bprom&group=programs&subgroup=gfindb

Estimation of the number of GlxI & GlxII enzymes per cell

The number of GlxI and GlxII molecules in a single cell can be estimated from the knowledge of the enzyme activity of the purified proteins, the activity in cell extracts and the weight of total cellular protein.

The purified GlxI enzyme exhibits a maximal activity of ~676 µmol · min^{-1}· mg^{-1} protein. The dimeric protein has molecular mass of ~30 kDa (Clugston *et al.*, 1998), thus 1 mol of dimeric GlxI can convert ~20280 mol of substrate per minute. The specific GlxI activity in *E. coli* extracts is ~0.016 µmol · min^{-1} · mg^{-1} total cell protein (MacLean *et al.*, 1998). Given the activity of the pure

protein, this equates to ~7.89 x 10^{-13} mol GlxI per mg of total cell protein. We can approximate that 1 mg total cell protein is equivalent to ~3.6 x 10^{9} cells based the following assumptions: OD_{650nm} of 1 = 1 x 10^{9} cells/ml, OD_{650nm} of 1 = 0.5 mg cell dry weight/ml (Elmore *et al*., 1990), protein content of cell dry weight: 55 % (Neidhardt & Umbarger, 1996). Therefore a single cell will have 2.17 x 10^{-22} mol of GlxI, which after consideration of the Avogadro constant (6.022 x 10^{23}) equates to an estimated number of GlxI molecules of ~130 dimers per cell.

The same calculations can be performed for the GlxII enzyme. The maximal activity of the purified enzyme is ~112 µmol · min^{-1} · mg^{-1} protein and the molecular mass is ~ 28.4 kDa (O'Young *et al.*, 2007). Thus 1 mol of pure protein can hydrolase ~3180 mol of substrate per minute. An approximation of the number of GlxII molecules from the activity in cell extracts requires further considerations. GlxII is a metallo-enzyme and it has been reported that the active site of the purified *E. coli* enzyme is loaded with zinc ions (O'Young *et al*., 2007). A study by Campos-Bermudez *et al* (2007) has shown that glyoxalase II from *Salmonella typhimurium* (78 % identity to *E. coli* GlxII; expressed in *E. coli*) is a metal promiscuous enzyme and can incorporate different metal ions depending on the availability in the medium, ultimately influencing the kinetic constants. A study within our group suggest a similar behaviour for the *E. coli* GlxII enzyme and the specific activity in extracts from cells grown in $K_{0.2}$ minimal medium and supplemented with 200 µM $ZnCl_2$ is ~0.03 µmol · min^{-1} · mg^{-1} total cell protein (Almeida, 2009). By relating this activity to the activity of the pure protein (zinc enzyme) one can estimate each cell to contain 2.62 x 10^{-21} mol GlxII and therefore ~1580 molecules.

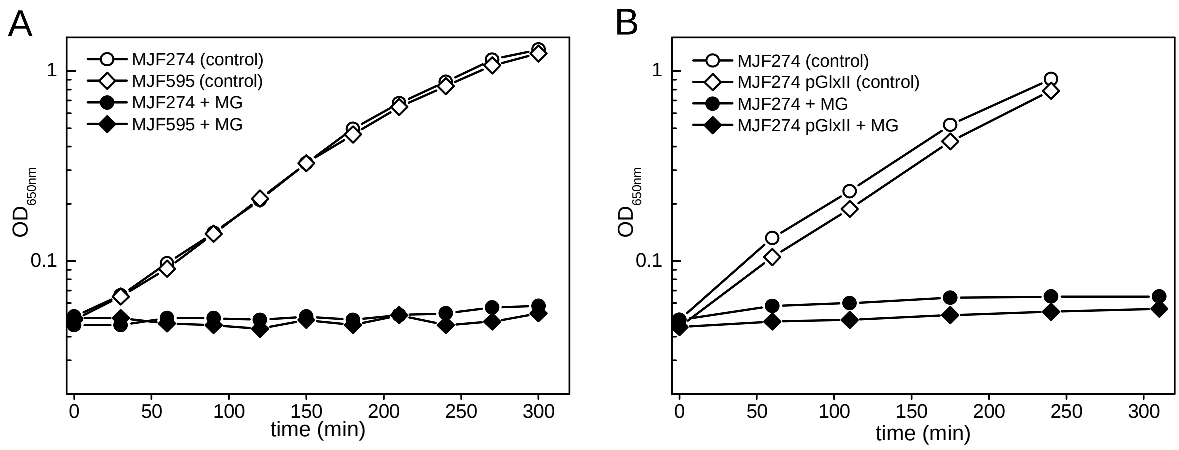

Fig. S1. Growth of strains deleted for *gloB* and over-expressing *gloB* are similar to the parent strain.

Growth of the *gloB* null mutant (**A**) and strain over-expressing *gloB* (pGlxII, **B**) are not affected. Cells from the parent and the Δ*gloB* mutant or over-expressing strains were grown overnight in K0.2 minimal media, diluted into fresh media and cultured to OD650nm of ~0.4. Cells were then diluted 10-fold into fresh media in the absence (controls) or presence of 0.7 mM MG. The data are representative of three independent replicates.

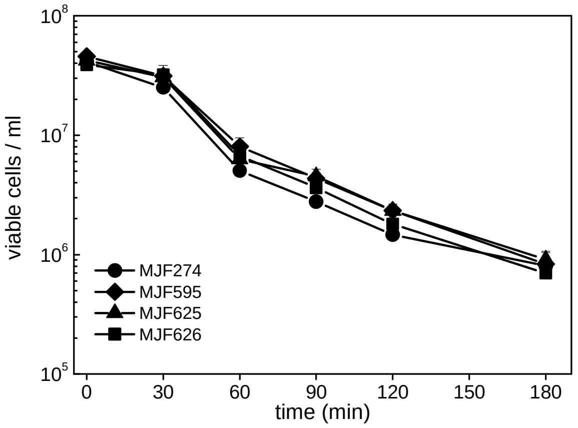

Fig. S2. Survival of *gloB* null mutant upon MG stress is not dependent on enzymes with minor SLG hydrolase activity.

Cells that lack either YeiG or FrmB in addition to GlxII are not more sensitive to MG stress than the single mutant lacking GlxII. Cells from the parent (◇), Δ*gloB* (◆), MJF625 (Δ*gloB*, Δ*yeiG*; ▲) and MJF626 (Δ*gloB*, Δ*frmB*; ■) were grown in K0.2 minimal media, exposed to 0.7 mM MG and viable cells enumerated exactly as for experiments presented in Fig. 2. The mean and standard deviation of three independent experiments are shown.

A

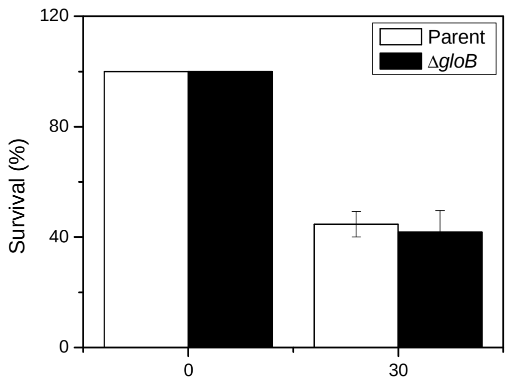

B

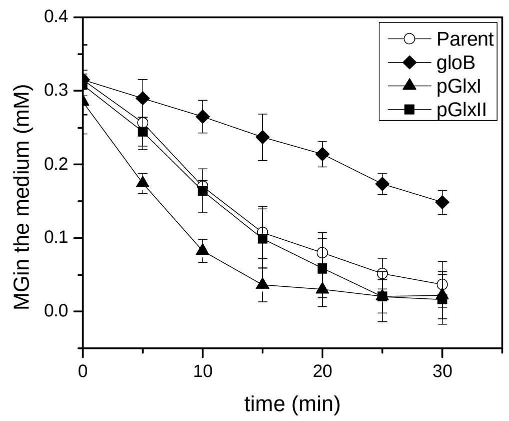

Fig. S3. High cell density survival and MG detoxification

A. The Δ*gloB* strain exhibits similar death kinetics to the parent strain upon exposure of high cell density cultures (OD650nm ~0.8) to 0.8 mM MG.

B. MG detoxification ability of cells from the parent (◇), Δ*gloB* mutant (◆), pGlxI (▲) and pGlxII (■) assayed at high cell density (OD650nm ~0.8).

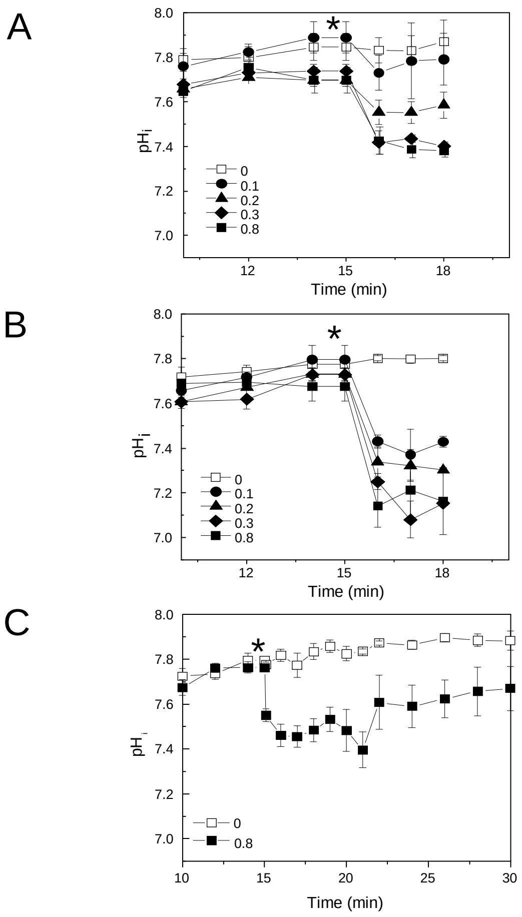

Fig. S4. The relationship between intracellular pH and MG
The intracellular pH was determined for the parent (**A**) and Δ*gloB* (**B**) cells treated with a range of MG concentrations (0 - 0.8 mM) in K0.2. The intracellular pH of parent cells was measured over 15 min after addition of 0.8 mM MG (**C**). * MG was added at t15 min thus the t15 min time point has been added as a duplicate of t14 min to illustrate the rapid kinetics of cytoplasm acidification. The data are mean ± s.e.m.
